# Read My Leads: Subject‐Specific RF Hazard Assessment and Mitigation for DBS Implants in MRI

**DOI:** 10.1002/mrm.70186

**Published:** 2025-11-24

**Authors:** Berk Silemek, Frank Seifert, Mevlüt Yalaz, Michael Höft, Günther Deuschl, Arzu Has Silemek, Rüdiger Brühl, Bernd Ittermann, Lukas Winter

**Affiliations:** ^1^ Physikalisch‐Technische Bundesanstalt (PTB) Braunschweig and Berlin Germany; ^2^ Department of Electrical and Information Engineering Christian‐Albrechts‐Universität zu Kiel Kiel Germany; ^3^ Department of Neurology Christian‐Albrechts‐Universität zu Kiel Kiel Germany; ^4^ Department of Neurology Cedars‐Sinai Medical Center Los Angeles California USA

**Keywords:** deep brain stimulation, impedance, implants, MR safety, RF heating, sensors

## Abstract

**Purpose:**

To develop and validate a framework for personalized, implant‐specific MRI safety assessments using feedback from commercial deep brain stimulation (DBS) systems. To further use this framework to suppress RF‐induced heating with minimum compromise in imaging performance.

**Methods:**

Two off‐the‐shelf DBS implantable pulse generators and a commercial 8‐electrode DBS lead were utilized for quantitative safety assessments. In controlled phantom experiments, (i) RF‐induced voltages on the DBS lead, and (ii) temperature‐dependent admittance/impedance changes in the tissue surrounding the lead's electrodes were quantified. This information was used to suppress implant‐related RF heating by calculating implant‐friendly imaging modes. Experimental conditions included excitations with different RF transmit coils (8‐channel 3 T and 7 T head coils, 2‐channel 3 T body coil), over 1000 different exposure scenarios, different implant configurations, and the use of external reference probes (E‐field and temperature) for validation. Imaging performance of the applied implant‐friendly mode was demonstrated in vivo on a 3 T scanner.

**Results:**

E‐fields and temperature rises around the tip electrodes could be robustly detected directly from the DBS lead. Both signals quantify the momentary patient hazard. Utilizing these measurements–recorded and wirelessly transmitted by the DBS system–tissue heating was reduced up to 99% for the same transmission power with comparable imaging performance to a conventional imaging mode.

**Conclusion:**

All the information needed for full in situ control of implant heating in MRI can be read directly from the DBS device. This approach would improve both patient safety and image quality while simultaneously reducing workload and responsibilities of the clinical personnel.

## 1 Introduction

Deep brain stimulation (DBS) [1, 2] is an essential medical treatment, increasingly employed to restore physiological function and manage debilitating neurological disorders. 56% of DBS indicated patients are estimated to need an MRI exam within 5 years of implantation [3]. From an MRI perspective, this is worrying [4] since the long metallic leads of DBS implants and many other active implantable medical devices (AIMD) pick up the RF fields from the MRI scanner and emit a “scattered” electric (E)‐field at the lead's tip electrodes in brain tissue which can exceed the “background” E‐field from the RF coil by orders of magnitude [5, 6]. Patients have been severely impaired by brain tissue burns near the electrodes [7, 8, 9, 10, 11], which triggered intensive research in the physics of implant heating and possible mitigation strategies [12, 13, 14, 15, 16, 17, 18, 19, 20, 21, 22, 23, 24, 25, 26, 27, 28, 29, 30, 31, 32, 33, 34].

MR compatibility, technically termed conditionality [35], of implants was pioneered for cardiac pacemakers [36], when the US Food and Drug Administration judged the case pressing enough to warrant a fast clinical approval for these devices [37]. Current MR conditionality assessments by implant manufacturers rely on hazard predictions from ex ante numerical simulations for a large variety of possible exposure scenarios, including varying anatomies, implant‐lead trajectories, and patient positions [38]. One manufacturer reports the simulation of 38 000 scan conditions and 10 million simulated patient scans [39]. Nevertheless, in clinical practice a significant curtailing of the permitted RF power is often imposed, either as a SAR or $B_{1,\mathrm{rms}}^{+}$ restriction [11], thus trading image quality for patient safety. Not only patients suffer from this situation, but also hospitals and their personnel since they have the sole responsibility and liability for implant safety. As a consequence, long and complex pre‐scan assessments need to be performed [40, 41] leading to substantial organizational challenges and costs [4] and significant delays and denials of MRI scans for implant carriers [3, 4, 40, 42, 43].

Here, we propose an alternative approach toward safe MRI scanning of patients carrying AIMD with long metallic leads: personalized measurements rather than generalized simulations. In previous work, it was investigated how small and inexpensive physical sensors attached to the implant tip can be utilized to detect and mitigate RF‐induced heating [44, 45, 46]. However, this required modifications to the implant lead and its electrodes, functionally the most critical area, which manufacturers are reluctant to implement. Now, we demonstrate that unmodified, commercial DBS implant components have the capability already built in to detect and quantify the momentary RF hazard: the implant itself is the sensor we need and even the electronics to transmit this information to an external receiver are already integrated [47, 48].

Phantom experiments were performed on a commercial 8‐electrode directional DBS lead and two different commercial implantable pulse generators (IPG) that were exposed to over 1000 different RF field configurations from a 3 T and a 7 T 8‐channel parallel transmit (pTx) head coils. External E‐field and temperature probes near the electrodes provided independent reference measures to confirm our approach. First, this work explores how the implant itself can be used to detect and quantify the momentary RF hazard for the patient. Next, it is investigated how such information can be exploited to control and mitigate the hazard. Finally, the practical feasibility of the mitigation approach is demonstrated in MRI experiments on a commercial 3 T MR scanner.

## 2 Methods

**FIGURE 1:**
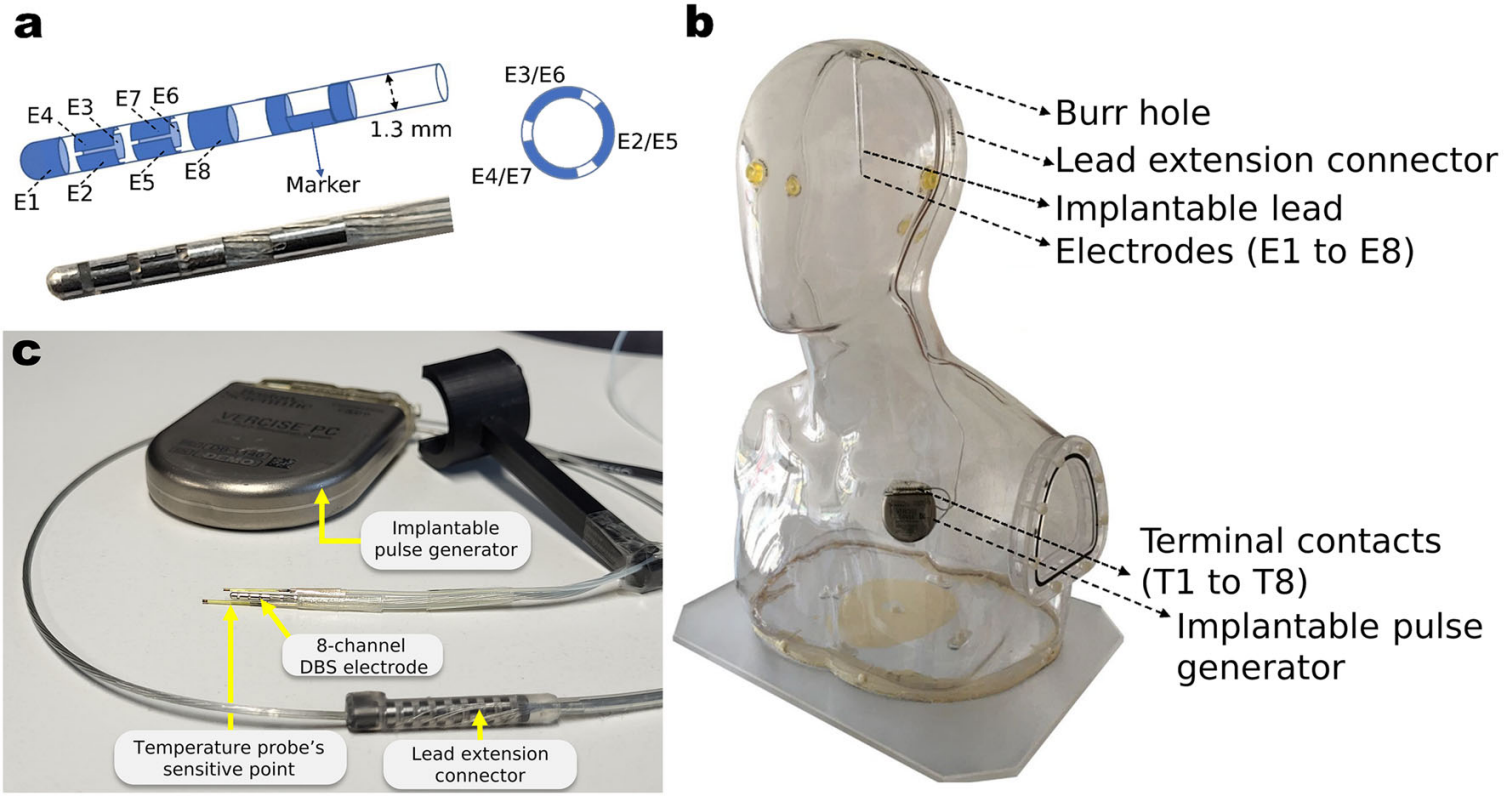
(a) Schematic and photograph of the tip end of the deep brain stimulator (DBS) lead carrying eight electrodes (E1–E8). (b) Photograph of the DBS system in a saline‐filled anthropomorphic phantom. The implant was realistically placed inside the phantom by an experienced DBS neurosurgeon [49]. (c) Photograph of the DBS implants' components used in the experiments including the lead extension connector and external temperature sensors.

Typical for modern DBS leads, the investigated commercial 8‐electrode implantable lead (Cartesia, Boston Scientific Corporation), as illustrated in Figure 1a, consists of eight conductive wires, carrying stimulation pulses from the terminal contacts T1–T8 at the IPG (Figure 1b) to the electrodes E1–E8 over a lead extension connector (Figure 1c) to the tip end in tissue. During an MRI experiment, the scanner transmits an RF field which is collected and concentrated by the wires in the lead (“antenna effect”). The scattered E‐field $\left(E_{S}\right)$ emanating from the electrodes can then cause excessive heating in the surrounding tissue. This constitutes the patient hazard, and the task is to quantify it in order to control it. Previous simulation work has shown that both $E_{S}$ (cause) and the temperature rise (consequence) in the vicinity of the lead electrodes are suitable and largely equivalent metrics for this purpose if they can be detected in situ [46].

**FIGURE 2:**
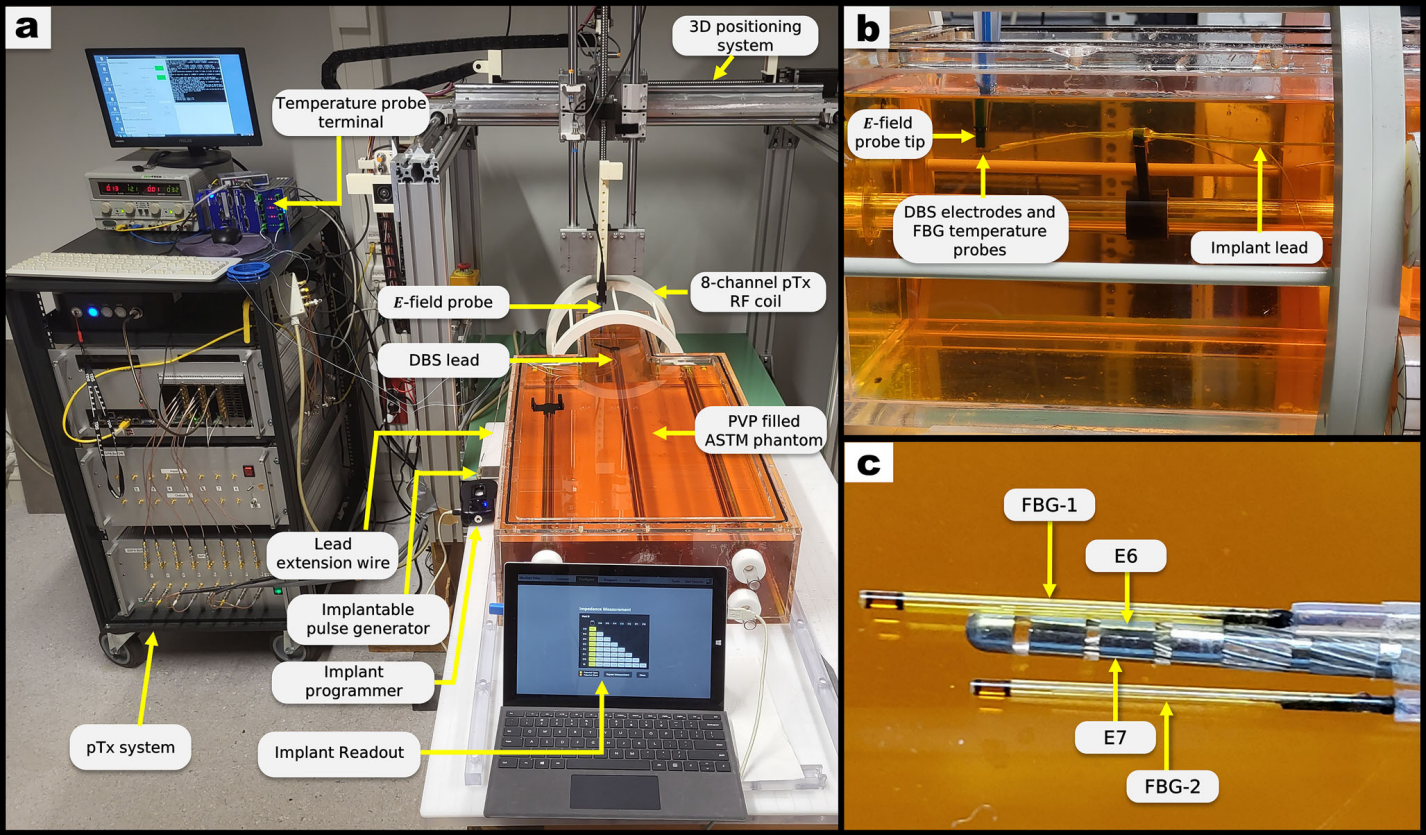
Experimental setup for the test bench experiments. (a) Polyvinylpyrrolidone (PVP) filled ASTM phantom with its head section inside the 8‐channel RF coil. (b) Side view of the implant lead with an E‐field probe at its tip (blue, coming from the top) and two fiber Bragg grating temperature probes all located within the RF coil and ASTM phantom. (c) DBS implant and electrode configuration with a detailed placement of the fiber Bragg grating (FBG) temperature sensors, the arrows point their sensitive location which is not at the tip.

The implant lead and interface were immersed in an American Society of Testing Materials (ASTM)‐phantom container [50] filled with polyvinylpyrrolidone (PVP) solution, with dielectric parameters ($\varepsilon_{r}$ = 50 and = 0.33 S/m at 128 MHz) closely matching the respective values for white brain matter [51, 52]. The phantom's head section was surrounded by either a commercial 3 T (RAPID Biomedical) or an open‐source 7 T [53] MRI head coil, each with eight transmit channels (see Figure 2a). The coil in use was hooked to an implant safety testbed [44] which allows to generate eight phase‐locked, continuous wave RF signals with freely selectable amplitudes and phases and up to 20 W per channel.

### 2.1 Detection of the Scattered E‐Field Around the Electrodes: The Auto‐Sense Signal

The scattered E‐field around the lead's tip generates a net RF voltage between any two lead electrodes. This voltage can be detected and, crucially, this can be done “remotely” at the terminal contacts in the pulse generator since all fields induced along the lead are nearly identical in all eight wires and cancel out very effectively. This RF voltage between two electrodes is henceforth referred to as the “auto‐sense” signal $\left(U_{\mathrm{AS}}\right)$. To detect $U_{\mathrm{AS}}$, which is not yet available from the commercial IPG, the proximal end of the lead was connected to a home‐built interface to read the RF voltage between electrodes. This signal was fed to the receiver of the testbed which provided the common time base for all experiments as well as synchronized input channels to record and digitize external probes for reference measurements. $U_{\mathrm{AS}}$ is recorded for 1000 random RF excitation vectors (pulse lengths 100 μs), each generating a unique electromagnetic field distribution inside the phantom. Simultaneously, $E_{S}$‐field components normal to the electrodes were measured in the phantom liquid in ∼2 mm distance from the lead by an external time‐domain E‐field probe (E1TDSz, SPEAG) as illustrated in Figure 2b.

For the more time‐consuming heating experiments, 21 of the previously used 1000 RF excitation vectors were selected, covering the full range of $E_{S}$ values. The temperature rise in PVP during 60 s of RF exposure was measured using two high‐resolution (∼2 mK) fiber Bragg grating (FBG) temperature sensors (FBG‐T8, imc test & measurement GmbH) with the sensitive spot in about 1 mm distance from electrode E1 (see Figure 2c).

### 2.2 Detection of the Temperature Around the Electrodes via Impedance Measurements

For a patient specific calibration of their stimulation pulses, modern DBS systems can measure the tissue impedance between any pair of electrodes on the fully implanted device [54, 55, 56]. This occurs via an external “programming unit” (see Figure 2a), provided by the manufacturer, and uses a wireless communication link to the IPG. This built‐in functionality of the off‐the‐shelf device was exploited to detect temperature changes in the tissue surrounding the electrodes using only existing and unmodified, manufacturer‐provided hardware. The implant lead was connected to a matching commercial IPG (Vercise Gevia, Boston Scientific Corporation) and the complete DBS including a 30‐cm lead extension was realistically placed within an anthropomorphic phantom by an experienced DBS neurosurgeon (see Figure 1b) [49]. Inter‐electrode impedances were read from the remote DBS programmer while the power supplied to the surrounding RF coil was varied. Each RF pulse was set to 5 s to accommodate for timing uncertainties due to manual triggering. Impedance changes $∆Z_{i,j}=Z_{i,j}^{\mathrm{RF}\;\mathrm{on}}-Z_{i,j}^{\mathrm{RF}\;\mathrm{off}}$ relative to measurements without RF were calculated for each of the 28 electrode combinations i,j of the directional lead.

To investigate the mechanism behind the RF induced impedance changes, at first experiments without RF exposure were performed. The implant lead was immersed 5 cm deep into a tube‐shaped glass container filled with 40 mL of PVP solution and this tube placed into a temperature‐controlled water bath (RE630G, Lauda). A calibrated PT‐100 temperature probe inserted 2 cm deep into the phantom liquid provided precise reference temperatures. To achieve the high temporal resolution and precise measurement control required for this characterization, the proximal terminals (contacts to the pulse generator) T1 and T8 belonging to electrodes E1 and E8 were connected to a precision LCR meter (ST2829C, Sourcetronic) to measure time‐dependent admittance values Y=1/Z at 1 kHz with a source current set to 2 mA. Admittance Y was chosen over impedance Z, here, since the conductivity of aqueous PVP solutions is known to increase approximately linearly with temperature [51] in the given range of interest compared to gelatin phantoms [57, 58]. Both bath and PVP temperatures (1 Hz), and admittances (2 Hz) were simultaneously recorded while the bath temperature was increased from 20°C to 45°C at a rate of 1 K per 15 min. To establish a calibration curve, the relationship between PVP temperature and admittance was modeled using ordinary least squares linear regression. To ensure steady‐state conditions and mitigate thermal lag, data points were generated by averaging measurements over a 2‐min window, beginning 12 min after the start of each thermal step.

In the next experiment, the implant lead was placed in the ASTM phantom, again, and exposed to 60 s of RF heating at different power levels while the temperature near the electrodes was measured with two FBG probes at a 5 Hz rate, and the admittance between two electrodes by the LCR meter at a 24 Hz rate. Both 3 and 7 T frequencies were applied using the aforementioned RF coils. Admittances for three different electrode pairs were investigated to check for possible electrode dependence.

### 2.3 Implant Coupling Matrix and Mitigation of RF Heating

If more than one RF transmitter is available—many modern 3 T MRIs have two, 5 T and 7 T systems typically have eight such “parallel transmit” (pTx) channels—the spatial distribution of the RF field can be manipulated [59, 60]. This allows reducing the RF coupling to the implant and calculating “implant‐friendly” scan modes with little sacrifice in image quality [6, 29, 45, 61].

For quantitative assessments of the implant‐related hazard, the aforementioned time domain E‐field sensor and two temperature probes (FBG‐1 and FBG‐2) were placed in the ASTM liquid near the electrodes (Figure 2c). The lead was connected to another IPG (Vercise PC, Boston Scientific Corporation) with a matching extension cable and the inter‐electrode impedance changes $∆Z_{i,j}{=Z}_{i,j}^{\mathrm{RF}\;\mathrm{on}}-Z_{i,j}^{\mathrm{RF}\;\mathrm{off}}$ in response to RF exposure were measured via the DBS programmer as described before. Since DBS system and RF transmitter are not synchronized in this type of experiment, 5 s long RF pulses were applied to allow for manual triggering of the IPG readout.

The coupling of the individual RF channels to a given pair of lead electrodes can be described by the so‐called sensor Q‐matrix ($Q_{S}$) [45, 46, 61], such that any RF safety parameter of interest X, here the impedance change, can be expressed as:

(1) $$X=u^{H}Q_{S}u,$$

where u is the complex voltage vector applied to the coil ports. To construct the complex, Hermitian $Q_{S}$ from real‐valued sensor data, $N^{2}$ measurements are required for an N‐channel pTx system:

(2) $$Q_{S,\mathrm{kl}}=\left\{\begin{array}{l} \left(X_{\mathrm{kl}}-X_{k}-X_{l}\right)+j\left(X_{\mathrm{kl}}^{†}-X_{k}-X_{l}\right)\;\text{for}\;k\neq l\;\text{and}\;k<l\\ \left(X_{\mathrm{kl}}-X_{k}-X_{l}\right)-j\left(X_{\mathrm{kl}}^{†}-X_{k}-X_{l}\right)\;\text{for}\;k\neq l\;\text{and}\;k>l\\ 2X_{k}\;\text{for}\;k=l \end{array}\right.,$$

where $X_{k}$ is the sensor reading for a single coil channel k, whereas $X_{\mathrm{kl}}$ and $X_{\mathrm{kl}}^{†}$ are the readings for two channels k,l transmitting in‐phase and with a π/2 phase difference, respectively. Recording $∆Z_{i,j}$ for all 64 exposure conditions provides the desired $Q_{S}$ for an 8‐channel RF coil.

### 2.4 MRI Experiments

For the MRI experiments, the DBS lead was immersed in an ASTM phantom and placed inside a 3 T MRI scanner (Cima.X, Siemens Healthineers). The T1 and T8 contacts were connected to a ∼3 m coaxial cable via the aforementioned home‐built adapter and a low‐pass filter (10 MHz cut‐off). The signal is then routed to the outside of the RF cabinet and fed to the LCR meter. An RF‐only sequence was applied with RMS voltages set at the scanner console ranging from 5.0 to 40.7 V. After 4 s of RF exposure, $Z_{1,8}$ was recorded. Next, the scanner's 2‐channel body coil was used to mitigate implant heating. The RF‐only sequence was run with four different excitation vectors to acquire $Q_{S}$ which is now a 2 × 2 matrix. Worst case and implant‐friendly “orthogonal projection” [44] modes were calculated from $Q_{S}$ and applied via the scanner's manual adjustments. RF‐induced impedance changes $∆Z_{1,8}$ were recorded during 3D GRE sequences (0.5 × 0.5 × 2.09 mm^3^, FOV = 256 mm^2^, TE/TR = 3.69/8.0 ms, 128 slices, TA = 9 min, FA = 20°) and the expected temperature rise calculated. For in vivo experiments, a healthy volunteer was imaged using both the conventional, circular polarization (CP) and the orthogonal projection mode from the previous phantom measurements. 3D T1‐weighted MPRAGE images were acquired using the following settings: 0.73 mm isotropic, FOV = 352 × 352 × 175 mm^3^, TE/TI/TR = 3.16/1160/2360 ms, 128 slices, TA = 7:23 min. All in vivo experiments were approved by our local ethics board and informed consent was obtained from the participant.

For comparison of the image quality with CP excitation versus orthogonal projection mode, the volumes of various brain regions were determined using an automated procedure in FreeSurfer [62]. The procedure included the removal of non‐brain tissues such as the skull, eyeballs, and skin to enable precise whole‐brain segmentation. Cortical surface reconstruction methods were applied to derive regional cortical volume measurements. Furthermore, each subject was registered to the Destrieux atlas [63] using spherical registration, following the removal of white matter residuals as part of the “autorecon” processing stages in FreeSurfer.

## 3 Results

### 3.1 Detection of the Scattered E‐Field Around the Electrodes: The Auto‐Sense Signal

**FIGURE 3:**
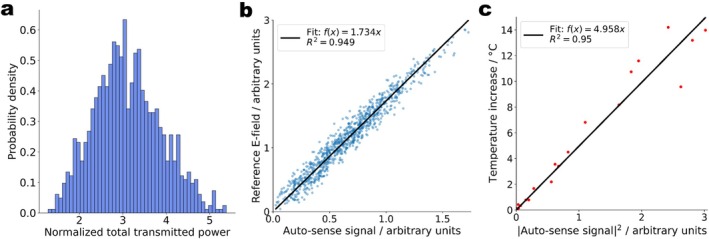
Detection of RF‐induced heating in DBS implants using the auto‐sense signal. (a) The distribution of the normalized total power at RF channels for the transmitted 1000 random RF excitation vectors. (b) Scatter plot of the reference E‐field in PVP phantom measured by an external probe near the electrodes versus auto‐sense signal measured between the lead's terminal contacts T1 and T8 for 1000 random RF excitation vectors. (c) Scatter plot of the temperature increase in the phantom after 60 s of RF exposure recorded by the external fiber Bragg grating temperature probes near the electrodes versus the square of the (T1, T8) auto‐sense signal.

The distribution of the normalized total power of 1000 random 8‐channel RF excitation vectors is shown in Figure 3a. A scatter plot of $U_{\mathrm{AS}}$ versus corresponding reference E‐field measured by the external field probe (Figure 3b) shows a linear correlation ($R^{2}=0.949$) of both quantities. This result is corroborated by the linear correlation ($R^{2}=0.95$) of $U_{\mathrm{AS}}^{2}$ with reference temperature measurements (Figure 3c). In summary, Figure 3 demonstrates that $U_{\mathrm{AS}}$, read directly from a commercial DBS lead, is a valid measure of the scattered E‐fields in the tissue surrounding the electrodes. $U_{\mathrm{AS}}$ thus quantifies the implant‐related RF hazard.

### 3.2 Temperature Effect on Impedance Measurements

**FIGURE 4:**
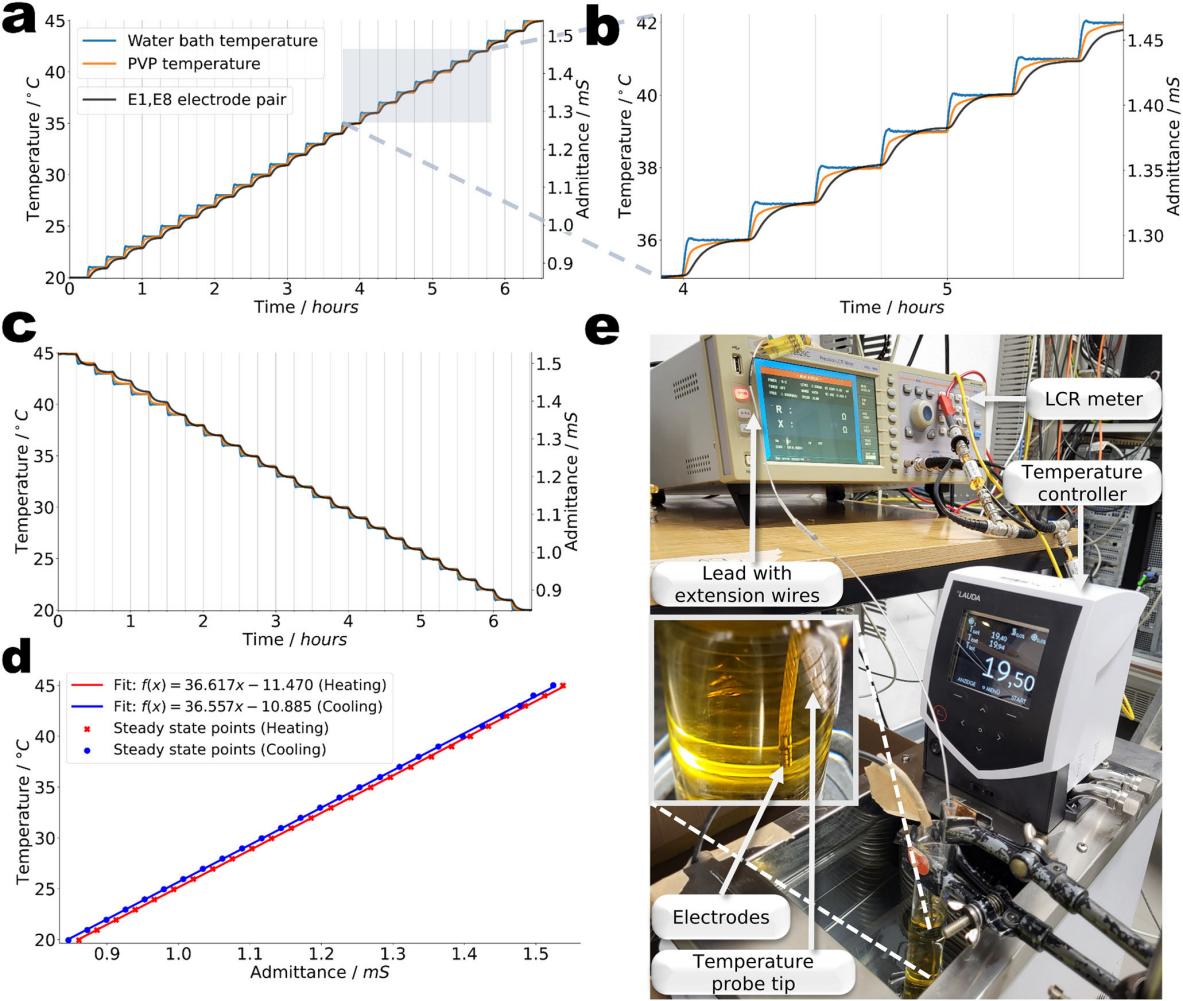
Simultaneous measurements of temperature and admittance (complex‐valued conductance) in a sample of the PVP phantom liquid. An LCR meter was connected to T1 and T8 of the implant lead, measuring the electrolyte's admittance between electrodes E1 and E8. The sample was kept in a temperature‐controlled bath whose temperature was varied in 1°C steps every 15 min. No RF was applied for this measurement. (a) Measured admittance, water bath temperature, and PVP temperature versus time within the heating cycles. (b) Detailed view from the data presented in a. (c) Measured quantities during the cooling cycle. (d) Admittance versus steady‐state temperature at the end of each temperature step. (e) Detailed view of the experimental setup.

The results of the heat‐bath experiments performed to investigate the temperature dependence of the inter‐electrode admittance Y in PVP independently of any RF exposure are shown in Figure 4, confirming a linear correspondence between admittance and temperature from 20°C to 45°C. During the temperature cycling both heating and cooling demonstrated a linear correlation ($R^{2}$ = 0.99) with comparable (slope deviation of 0.0016%) fitting values (Figure 4d). These results demonstrate that the electrolyte's temperature has a substantial and well measurable effect on the measured admittance. From Figure 4c, a relative admittance change (∆Y) of 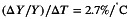 is obtained which is comparable with the literature value for our PVP formulation [51]. The absolute sensitivity for the E1, E8 electrode pair is 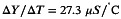.

### 3.3 Detection of the Temperature Around the Electrodes via Admittance Measurements

**FIGURE 5:**
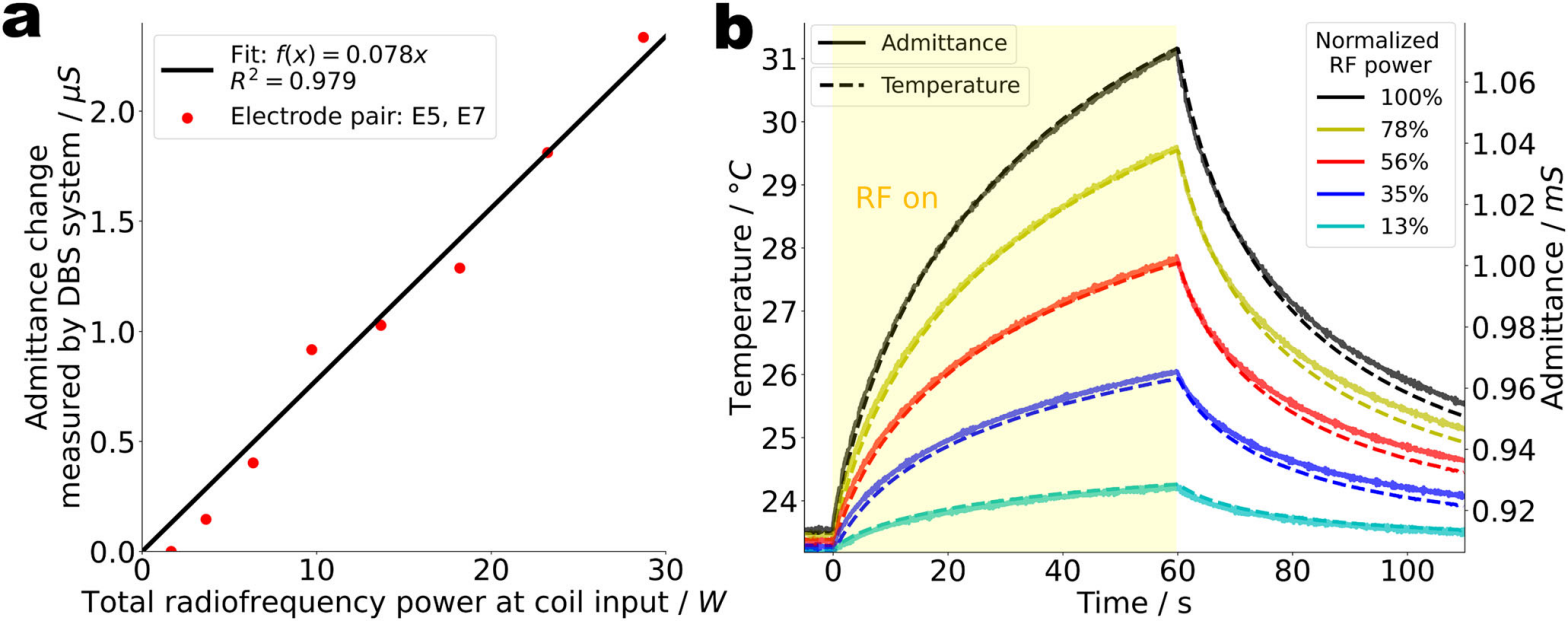
Detection of RF‐induced heating in DBS implants using admittance measurements (a) Admittance change immediately after RF exposure versus total transmit power applied to a commercial 3 T RF coil. Impedance values for the electrode pair E5, E7 were measured and wirelessly transmitted by the commercial DBS implant and then converted to admittances. A linear correlation is observed. (b) Temperature near the implant tip, measured by a fiber Bragg grating temperature probe, and admittance between terminal contacts T1 and T8, measured by the LCR meter, over time. RF heating at different power levels was applied from t=0–60s. Note that the figure shows absolute values for both admittances and temperatures. The traces are segments of one consecutive measurement over 35 min, shifted in time to have the heating periods aligned. The vertical scales were chosen such that the temperature and admittance traces coincided at the beginning of the first and at the end of the last RF exposure period (greenish and black curves, respectively). No further corrections were applied.

Using the complete commercial DBS system, that is, the lead connected to the IPG, a linear correlation (R^2^ = 0.98) is observed between the RF power transmitted to the coil and the admittance change ∆Y measured by the implant at one electrode pair as shown in Figure 5a. The increasing admittance changes reflect an increasing conductivity of the electrolyte between the electrodes in response to the RF‐induced temperature rise. The DBS system has a reported precision of ±1 Ω. For a typical 1‐kΩ tissue impedance and a temperature sensitivity of ∼2%/°C [64, 65], a 1‐Ω change corresponds to a temperature change of ∼0.05°C.

That impedance changes do indeed detect temperature changes is confirmed by a control experiment (Figure 5b) using the more precise and faster responding LCR meter instead of the DBS programmer. When the implant lead in the phantom is exposed to 60 s of RF heating at different power levels, the temperature near the electrodes, measured with external FBG temperature probes, and the admittance between two electrodes, measured by the LCR meter, vary in almost perfect synchronicity. Note that the admittance shows no sudden jumps when the RF is turned on or off. This proves that there is no *direct* RF effect, neither by the background field from the coil nor by the scattered field in the liquid. The admittance readings reflect the temperature and nothing but the temperature in the tissue equivalent liquid.

**FIGURE 6:**
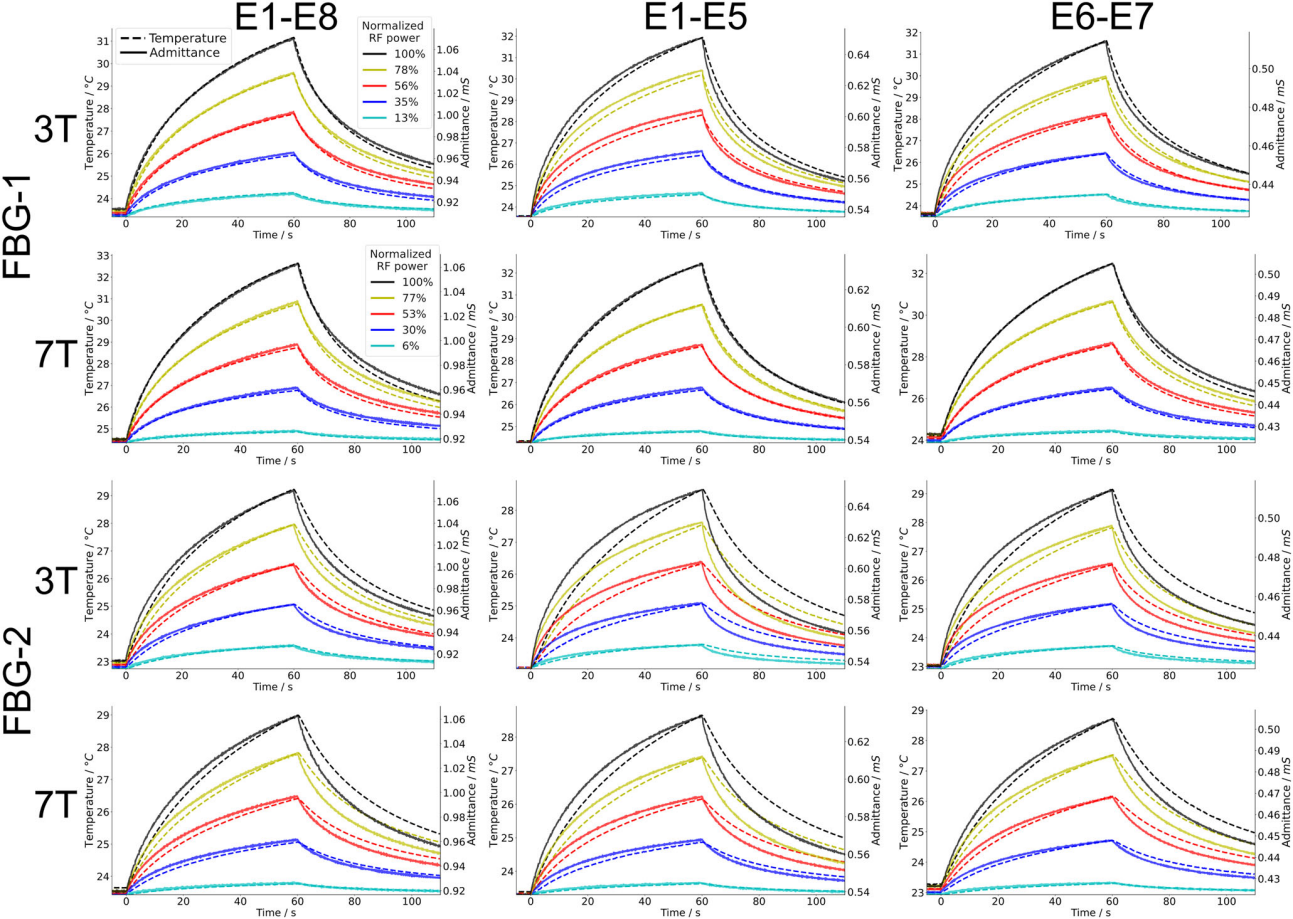
RF‐induced heating and admittance measurements using three different electrode combinations and two different RF coils for frequencies 128 MHz (3 T) and 298 MHz (7 T). For each combination, RF was applied for 60 s at five different power levels. Admittances were measured by an LCR meter connected to the respective terminal contacts (T1‐T8, etc.) of the lead, temperatures by two fiber Bragg grating (FBG) temperature probes FBG‐1 and FBG‐2 with FBG‐1 located slightly closer to the electrodes (see Figure 2c). The admittances from the two top rows are repeated in the bottom rows. Admittances probe the phantom liquid connecting the respective electrodes; their absolute values vary because of different electrode geometries and possibly different temperatures in the connecting bulk PVP volumes.

These results were reproduced for different electrode pairs, FBG probes and RF frequencies as shown in Figure 6. RF heating can reliably be detected by ∆Y measurements and this determination is independent of how the temperature change was created. It is thus independent of the applied RF coil or frequency being used, making ∆Y a generally applicable measurand for implant safety assessments.

The two bottom rows in Figure 6 repeat the admittance data from the two top rows but now compared to the other temperature sensor. Although both sensor readings correlate linearly ($R^{2}=0.98$), the slightly larger distance between electrodes and FBG‐2, see Figure 2c, results in a noticeable slower response and also lower peak temperatures, compared to FBG‐1.

### 3.4 Mitigation of RF Heating Using Parallel Transmission

The results so far demonstrate that either of the described measurands ($U_{\mathrm{AS}}$ or ∆Y) *quantifies* the momentary RF hazard for an implant‐carrying patient, specific for the given subject, implant trajectory, and scan conditions. Now we turn to the question of how to exploit such information to reduce RF‐induced heating and this can be done most effectively by using multiple RF channels, that is, pTx.

**FIGURE 7:**
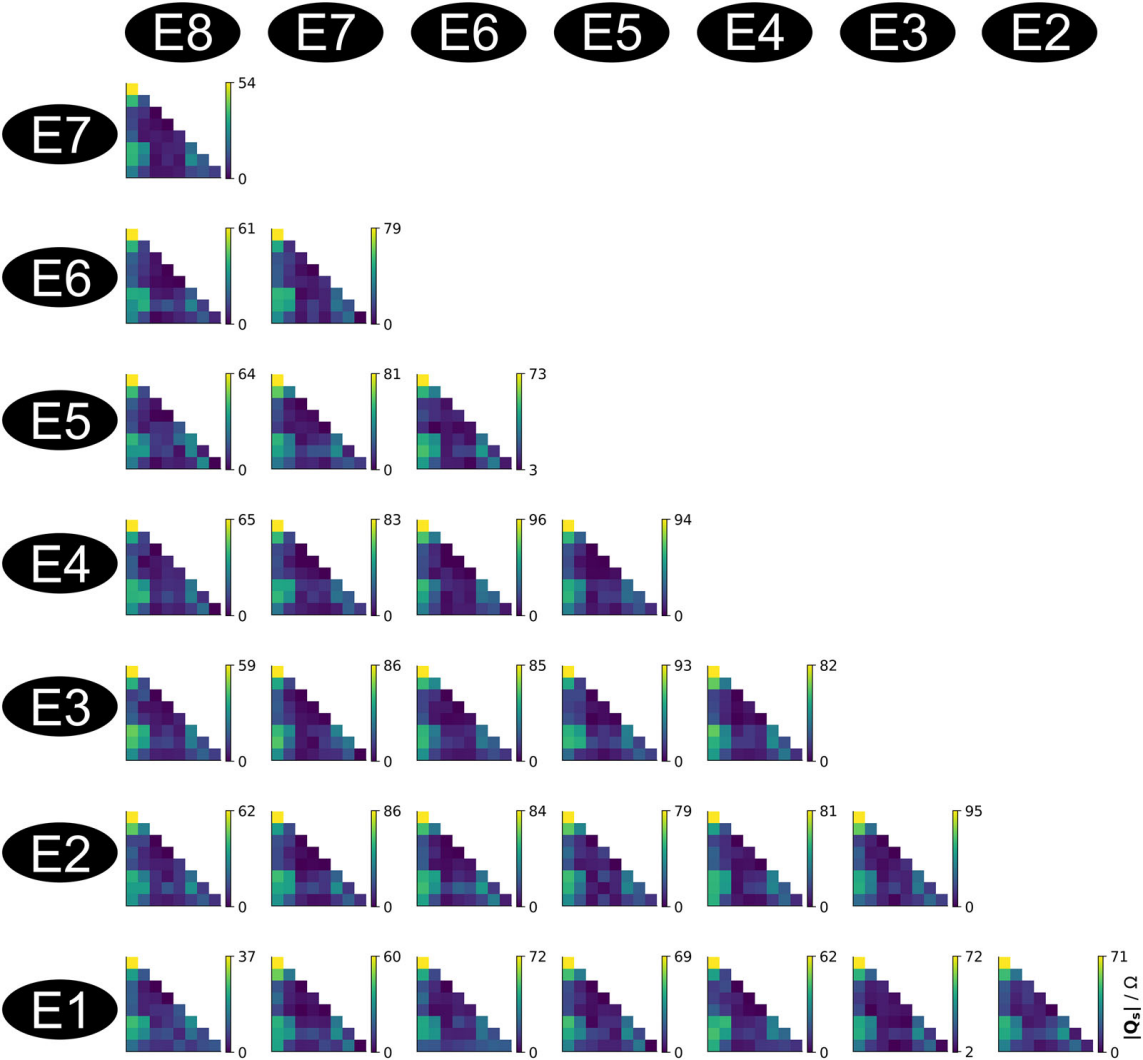
RF coupling derived from built‐in impedance measurements of the full DBS system. Testbed measurements of the sensor‐Q‐matrices ($Q_{S}$) for the 8‐channel 3 T RF coil are shown for all 28 combinations of the 8 lead electrodes. The color bars are individually scaled to the respective maximum element indicated at each bar (in units of ohm).

Figure 7 depicts the normalized sensor Q matrices, $Q_{S}$, for the 8‐channel pTx head coil at 3 T for all 28 electrode combinations of the 8‐electrode directional DBS lead. Their information content is largely redundant, however, since the RF coupling to the implant occurs along the long wires connecting electrodes and IPG terminals and the spatial separation of these wires is much smaller than the RF wavelength. The induced RF currents are always identical on all eight wires, therefore, and the relative spatial distribution of both $E_{S}$ and the temperature rise around the electrodes are always the same, independent of the RF mode. Different electrode combinations can be used to consistently derive the same coupling matrix, except for a scaling factor, improving the robustness of the method.

**FIGURE 8:**
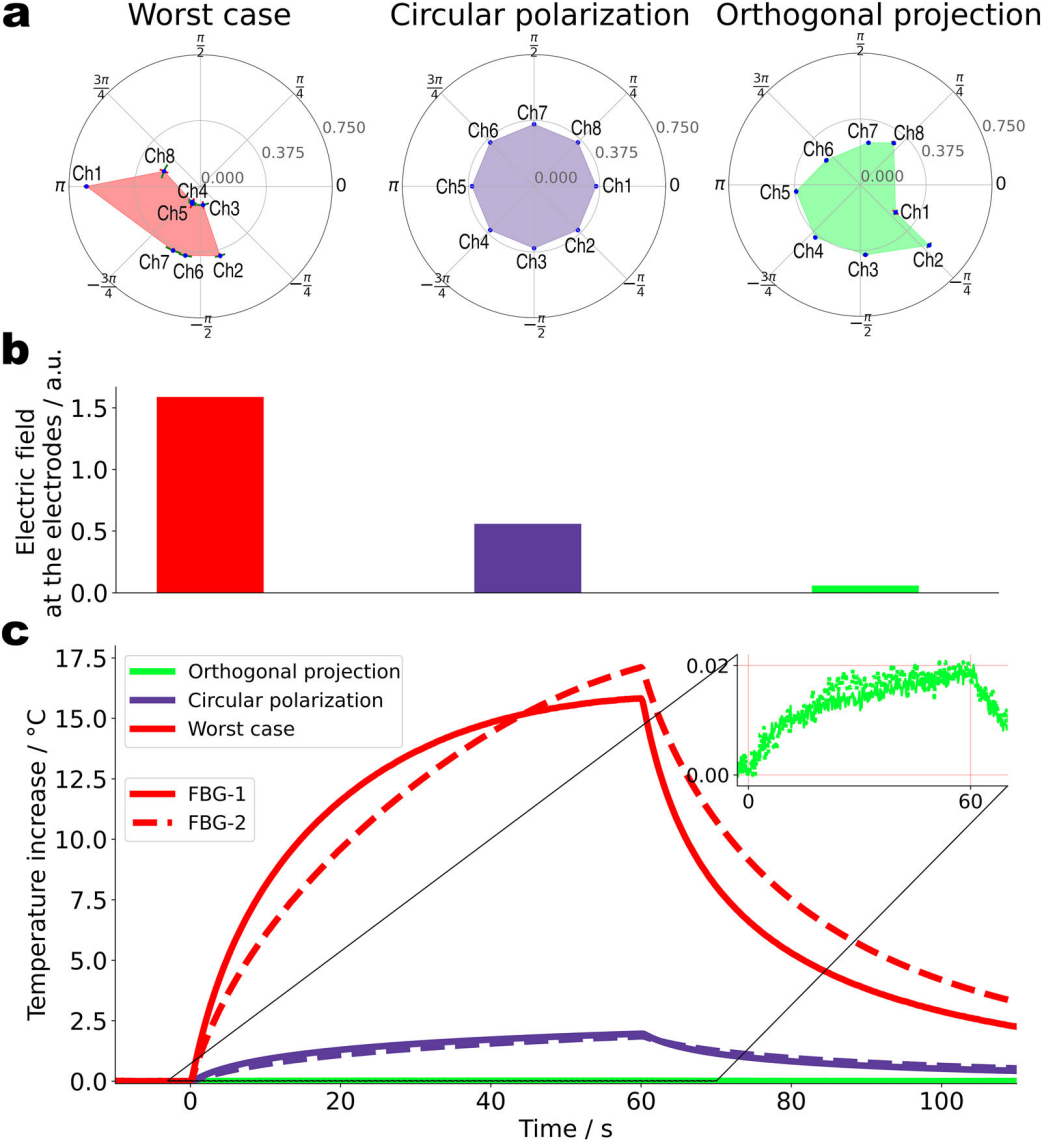
Using parallel transmission to mitigate implant heating. (a) Comparison of three RF transmission modes: Worst case (WC), circular polarization (CP), and the orthogonal projection (OP). The OP mode is calculated on‐the‐fly based on the measured $Q_{S}$. (b) E‐fields near the electrodes measured by a field probe in the phantom liquid when all three modes are transmitted with the same RF power. The OP mode creates 10 times lower, and the WC mode 3 times higher E‐>fields compared to the CP mode. (c) Temperature rise measured by two high‐resolution fiber Bragg grating sensors near the electrodes during 60 s of RF exposure for all three modes. The worst case mode demonstrates significant heating up to 17.1°C followed by the CP mode with 2.0°C. The OP mode barely shows temperature increase (0.02°C) as highlighted in the zoomed inset.

With $Q_{S}$, the implant heating properties of all possible pTx modes are known and tailored modes for optimum image quality under the constraint of safe scan conditions are determined. Three exemplary modes are depicted in Figure 8a, all transmitted with the same RF power. The reference is the CP mode, the conventional mode for head imaging in 3 T MRI, while the worst case and orthogonal projection mode were constructed by utilizing $Q_{S}$ [44]. The worst case mode maximizes RF coupling to the implant, it allows to calculate the maximum risk to the patient. Orthogonal projection, in contrast, is an “implant‐friendly” imaging mode, designed to inherit good image quality from the CP mode while avoiding strongly coupling RF channels.

The reference E‐fields measured with the external probe are shown in Figure 8b. In implant‐friendly mode, the E‐field is 10 times lower and in the worst case 3 times higher, compared to the CP mode. Temperature measurements (Figure 8c) confirm these results. After 1 min of RF exposure at identical RF power levels (16.85±0.45 W), the temperature near the electrodes increased by 2.0°C for the CP mode, 17.1°C for the worst‐case mode, but only 0.02°C for the orthogonal projection mode. Implant heating was reduced by a factor of ∼100, therefore, just by utilizing the built‐in impedance measurements of the implant to switch the MR scanner's transmit mode from conventional to implant‐friendly.

### 3.5 MRI Experiments

**FIGURE 9:**
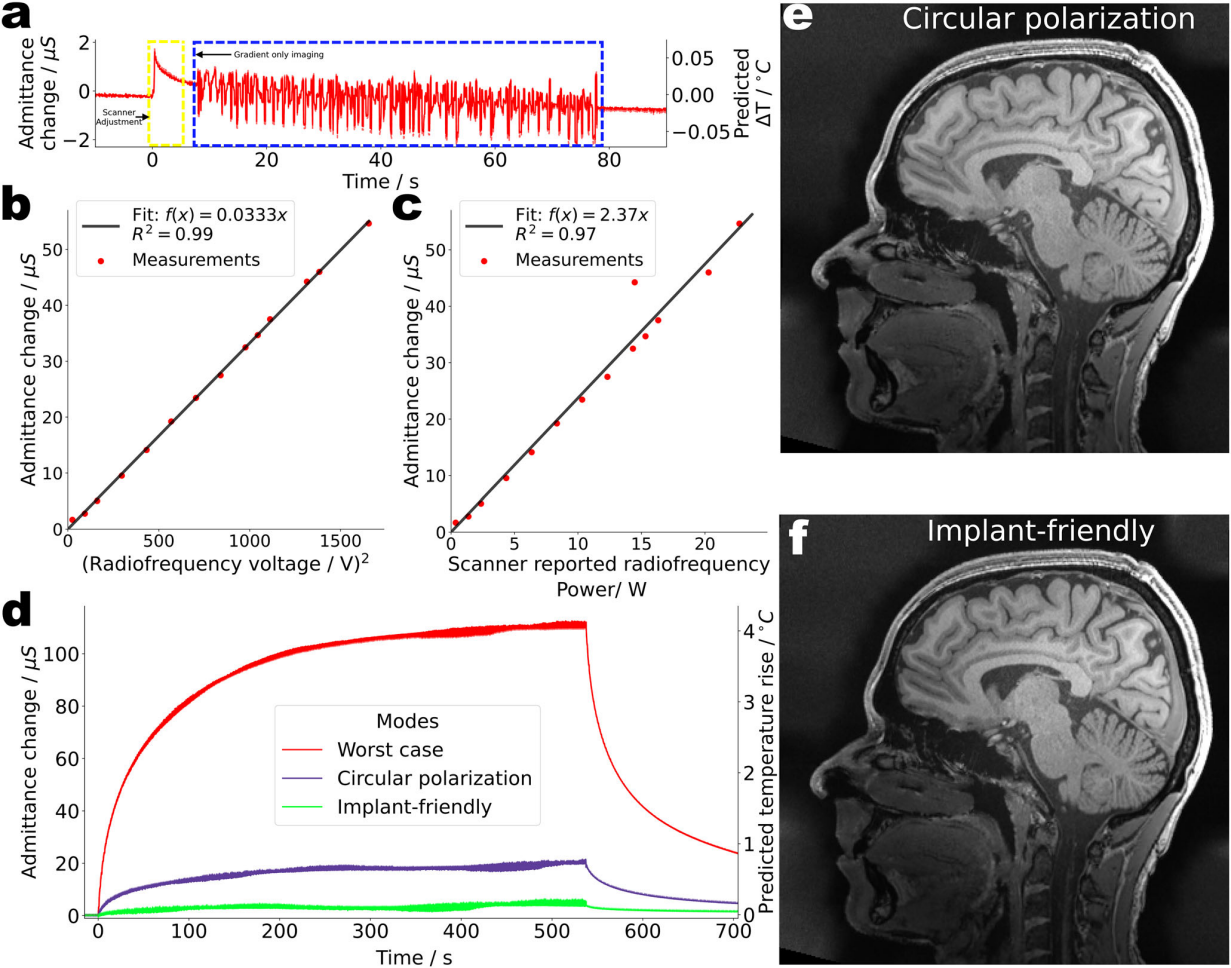
Mitigation of implant heating in a commercial 3 T scanner. (a) Detected admittance and predicted temperature changes during scanner adjustments and gradient‐only imaging are shown. (b) Measured admittance changes of the DBS electrodes in an ASTM phantom inside the scanner's body coil for different RF voltages set at the console. (c) Scanner‐reported average RF power and corresponding admittance changes between E1 and E8. (d) Admittance changes and predicted temperature rises during a ∼9‐min‐long MRI sequence using different transmission modes to drive the scanner's 2‐channel body coil. Compared to the circular polarization mode at the same RF power, implant heating is reduced by a factor of 5.0 in the implant‐friendly mode and increased by a factor of 5.5 under worst‐case excitation. (e) In vivo images of a healthy volunteer using the circular polarization and (f), the implant‐friendly imaging mode from (panel d), showing comparable image quality (structural similarity index measure: SSIM = 0.999).

The practical feasibility of the proposed procedures is investigated by transferring the experiments from the testbed to a commercial 3 T MRI scanner. The admittance changes between two electrodes (E1, E8) of the DBS lead in a phantom were measured at their corresponding terminal contacts (T1, T8). The accuracy of admittance readings under MR conditions is illustrated in Figure 9a. The RF‐only scanner adjustment pulses result in an admittance change of 1.7 μS corresponding to a temperature change of 0.066°C while the noise without RF corresponds to a temperature error of ±0.002°C. Consequently, the assessment of RF induced heating in the implant can effectively be performed at very low power levels with negligible implant heating. With imaging gradients but no RF (Figure 9a), the induced noise levels are ∆Y=±1μS corresponding to $∆T=\pm 0.04^{{}^{\circ}}C$, suggesting that sensitive temperature monitoring is feasible even under imaging conditions and without any attempt to filter out the gradient noise.

Figure 9b shows that ∆Y correlates quadratically with the transmitted RF voltage and linearly (Figure 9c) with the average transmitted power, both as reported by the scanner. To reproduce the mitigation experiments, the scanner's built‐in 2‐channel RF body coil was used to measure $\;Q_{S}$ and calculate the aforementioned transmission modes. For each mode, the admittance changes during a GRE sequence are displayed in Figure 9d, corresponding to temperature rises of 4.10°C, 0.75°C and 0.15°C for the worst case, CP and implant‐friendly mode, respectively. Again, the implant‐friendly imaging substantially reduces RF heating.

To illuminate the image quality of the implant‐friendly mode, the RF parameters determined above for an “implant‐carrying phantom” were used to acquire in vivo images from a healthy volunteer (Figure 9e,f). The mitigation‐mode image compares well with the CP reference image (structural similarity index measure SSIM = 0.999). A quantitative comparison by applying a standard neuroscience analysis to the full 3D imaging datasets, namely the parcellation of the cerebral cortex, is summarized in Figure S1. The independently calculated volumes of various brain structures agree well for both acquisition modes.

## 4 Discussion

The presented findings suggest a solution for arguably today's biggest and most complex MR safety problem: patients with AIMD. If implemented, improved safety, better image quality, and simplified clinical workflows can be expected for implant‐carrying patients. To validate the suggested approach, measurements were performed involving off‐the‐shelf DBS components, different phantom setups and implant lead routings, as well as RF coils for different MRI field strengths. Over 1000 different pTx excitations, each representing a unique electromagnetic field configuration, were tested using both a dedicated testbed and a commercial 3 T MRI scanner, demonstrating the method's robustness in detecting and mitigating RF hazards.

### 4.1 Auto‐Sense and ∆Y as Hazard Sensors

Two independent measures for the patient hazard were found and evaluated: the auto‐sense signal $U_{\mathrm{AS}}$ probing the net RF E‐field emanating from the tip electrodes, and admittance change, ∆Y, correlating with the local temperature rise near the electrodes. Either signal has the potential for a disruptive change in implant safety management. Both allow to quantify the RF hazard for an implant‐carrying patient in situ, specific for the given subject anatomy, implant trajectory, and scan conditions. Instead of millions of possible exposure scenarios only the momentarily given one is analyzed. This eliminates the need for large safety factors which are so‐far required due to the accumulated uncertainties when assessing the complex interactions of implant, RF coil, and patient by modeling [37].

Both signals average over the volume carrying the exploited information, that is, either the scattered E‐field or the low‐frequency test current for impedance measurements. This volume averaging is an advantage, since both E‐field and temperature vary drastically on sub‐mm length scales in the immediate vicinity of a metallic implant, which limits the reproducibility and the information content of all single‐point measurements in safety assessments. Volume measurements are more robust, for example, against minor positional variations, and thus provide more meaningful information in quantifying the risk for the patient.

Conceptually, both auto‐sense and ∆Y provide point‐sensor measurements, they characterize the $E_{S}$ or ∆T distribution by one single value. The choice of a particular electrode combination defines the “sensor position”, that is, the location of the probing volume, relative to the implant tip. All combinations provide redundant information; they only differ in a global scaling factor. Ultimately, the only relevant metric for implant safety is tissue damage, however, and only extensive numerical simulations can provide the link from sensor reading at a given position to the resulting tissue damage. Please note that this comment also applies to all existing implant safety assessments [38].

### 4.2 Technical Integration

We stress our use of unmodified, existing DBS components to underscore that technically the integration of the presented approaches into commercial devices should be a relatively small step. For practically useful implementations, changes to existing devices are nevertheless required. Presently, wireless communication between the IPG and programming unit is often disabled when the device is in “MRI mode.” Wireless communication from inside the scanner is routinely used for other devices [66], however, for example, ECG electrodes for cardiac triggering [67], and was also demonstrated for mock DBS systems [19, 61], so it is certainly possible if there is sufficient incentive to do it. For auto‐sense, the required hardware modifications to the IPG would likely be minor, as the necessary components such as ADCs, RMS detectors, RF switches, and filters are either already present in modern implants or are small, low‐power, and straightforward to integrate [61].

A routine exploitation of the ∆Y approach requires device modifications, as well. Manually reading impedance changes from the programming unit is too slow and error prone in a clinical setting. A sequence of fast ∆Y readings for all electrode combinations, triggerable from the programming unit, must be available when the device is in MRI mode. Unlike previously reported techniques [19, 45, 61], neither auto‐sense nor ∆Y require any modification of the DBS lead or the IPG readout circuitry themselves.

### 4.3 Auto‐Sense

From a pure physics perspective, auto‐sense has an advantage over ∆Y measurements. An RF E‐field is a robustly measurable quantity [38, 50, 68]. At MRI frequencies, the electrical response of biological tissue is dominated by its permittivity while conductivity has only a smaller, indirect effect via absorption [69]. Unlike ∆Y or local SAR which are both directly proportional to σ the E‐field is rather robust, therefore, against variations of the local tissue conductivity, whether between subjects or longitudinally within one subject.

For a practical implementation, an implant manufacturer must perform both measurements in a tissue‐mimicking phantom like in Figure 3 and numerical simulations in anatomical voxel models [46, 61] to obtain the detailed E‐field, temperature, and tissue‐damage distribution for the in vivo case. This combination allows to calibrate the auto‐sense signal in terms of the patient hazard. For a given MR field strength, this calibration depends only on the implant itself but not on patient anatomy or exposure conditions (MR scanner model, RF coil type, MR protocol, patient position or posture) [45]. The calibration can be done already at the device‐development stage, therefore, and stored in the pulse generator [61].

Merits and limitations of the auto‐sense approach are best discussed in comparison to the currently prevailing state‐of‐the‐art in assessing the MRI safety of AIMDs with long leads, the transfer function concept. Both approaches follow Tier 3 of ISO 10974 [38] and are conceptually equivalent, therefore. Tier 3 neglects any long‐range perturbations of the background E‐field ($E_{\mathrm{BG}}$) by the presence of the implant. The scattered field $E_{S}$ is the implant's linear response to $E_{\mathrm{BG}}$ and can be factorized as [5].

(3) $$E_{s}\left(\vec{r},E_{\mathrm{BG}}\right)=E_{1}\left(\vec{r}\right)\alpha \left(E_{\mathrm{BG}}\right),$$

where $E_{1}\left(\vec{r}\right)$ is the spatial distribution function of $E_{s}$ per unit $\alpha .E_{1}\left(\vec{r}\right)$ is an implant property, it can only be obtained by simulations but this needs to be done only once. $\alpha \left(E_{\mathrm{BG}}\right)$ is the excitation strength determined by the coupling of a given $E_{\mathrm{BG}}$ to a given implant. In the transfer function approach, $\alpha =\int_{0}^{L}S_{1}\left(\tau \right)E_{\mathrm{BG},\tan}\left(\tau \right)d\tau$, that is, the integral of the tangential $E_{\mathrm{BG}}$ component along the lead trajectory, is weighted by the implant's normalized transfer function $S_{1}$. The latter is an implant property, determined by ex situ measurements or simulations; $E_{\mathrm{BG}}$ and the implant trajectory must be known. In the auto‐sense approach, $\alpha ={\mathrm{kU}}_{\mathrm{AS}}$ is directly given by the auto‐sense signal. The sensitivity factor k depends on the selected electrode combination and can be determined as part of the $E_{1}\left(\vec{r}\right)$ simulations. Auto‐sense has no conceptual advantage over the transfer function approach, both are Tier 3, but a huge practical one, as it is a fast in situ measurement and no further knowledge about $E_{\mathrm{BG}}$, patient anatomy, or implant trajectory is required.

### 4.4 Temperature Measurements via ∆Y

Compared to auto‐sense, our second approach the ∆Y method is more affected by the tissue properties near and between the electrodes, like bulk conductivities or resistive and capacitive effects at the electrode‐tissue interfaces [55]. Inter‐subject differences of the latter interface effects should largely cancel out when only temperature‐induced admittance changes are considered. Studies on RF ablation and ex vivo tissue measurements consistently report a conductivity change of 1.5%/°C–2.0%/°C, confirming that while tissue‐specific the effect is predictable [64, 65]. A calibration of ∆Y in terms of the local temperature increase in the brain requires a combination of simulations and in vivo measurements. This mandates a clinical study on a sufficiently large patient cohort, which is costly, time consuming, and introduces new uncertainties. The benefits of ∆Y measurements are: (i) this feature is already implemented in many devices, and (ii) temperature rise is closer to the ultimate quantity of interest, tissue damage. Even less precise temperature information may, therefore, still be a better tissue‐damage predictor than a more precisely known E‐field.

Ultimately, implant manufacturers must implement either approach; they must decide which one complies best with their specific boundary conditions.

### 4.5 Clinical Implementation

Today, AIMD manufacturers store predetermined limit values for a scanner‐reported RF measure, $B_{1,\mathrm{rms}}^{+}$ or SAR, in the device documentation. MRI operators are responsible for knowing and obeying these limits. In the sensor‐based approaches proposed here, the manufacturers would pre‐determine limit values for $U_{\mathrm{AS}}$ or ∆Y and store them in the IPG or the programming unit. When the operator runs a short test sequence with known $B_{1,\mathrm{rms}}^{+}$, the programmer would compute and display a subject and exposure specific $B_{1,\mathrm{rms}}^{+}$ limit. So far, the operator would still be responsible for obeying this limit, while the goal must be direct communication between AIMD and MR scanner such that an implant‐unsafe sequence is automatically detected and rejected.

### 4.6 Mitigation and pTx

Scanners with parallel‐transmit RF systems offer the additional option of an on‐the‐fly optimization of the RF settings. At unchanged power levels in implant‐friendly mode, RF heating was reduced by 99% in testbed experiments using an 8‐channel transmit coil and still by 80% in MR experiments with 2‐channel excitation, a process which required only four measurements and took approximately 10 s. As seen before [20, 28], more transmit channels and hence more degrees of freedom to shape the electromagnetic field provide better heating suppression. In vivo experiments on a 3 T MRI scanner showed no relevant loss in image quality using the implant‐friendly mode.

### 4.7 Limitations

As a limitation of the current work, only DBS components from a single manufacturer were investigated. There is no proof that similar data can be obtained from other device models or other active implants such as spinal cord stimulators, but it would be unreasonable to assume that it cannot. Most measurements were done in vitro with a testbed in the laboratory, and not in vivo. The use of phantoms with tissue surrogates is standard practice in implant‐safety assessments; however, theoretically underpinned [70] and prescribed in relevant standards [38, 50] and FDA guidelines [35]. The use of the safety testbed simplifies and accelerates the experiments but does not change the physics. It has been shown previously [44, 45] and it has been shown here that the testbed results are equivalent to MR scanner experiments.

In our manual implementation of the ∆Y method it took several minutes to acquire all 64 impedance measurements to determine the $Q_{s}$ for an 8‐channel coil. This was due to the lack of communication between the RF transmitter and the DBS system and can be addressed with automation. With an integrated system, for comparison, temperature‐based and E‐field‐based $Q_{s}$ acquisitions have been performed in one minute [45] and 7.5 ms [61], respectively. For commercial 3 T systems equipped with a 2‐channel body coil, $Q_{S}$ acquisition requires only four measurements, which is feasible within seconds. If implemented, the acquisition times both for ∆Y and auto‐sense allow an integration of these measurements before, after or even during the MR sequence to enable continuous monitoring of implant RF safety without significantly prolonging clinical protocols.

## 5 Conclusion

In summary, critical information on the implant‐related safety hazard in MRI was read directly from off‐the‐shelf DBS components in two different ways. Measures of either the scattered E‐field or the temperature rise around the lead electrodes can be acquired in the pulse generator, compared to a predetermined safety limit, and wirelessly transmitted to an external device. Based on this information, any MR scanner can be adjusted to implant‐safe RF settings. An implant safety concept in MRI, based on suitable AIMDs communicating with the MRI system is desirable and technically within reach. The concept is not restricted to DBS systems and has the potential to significantly improve patient safety, enhance imaging performance, and streamline clinical workflows.

## Supporting information


**Figure S1:** Parcellation of the cortex using conventional and implant‐friendly imaging. The brain region volumes are calculated based on Destrieux atlas using implant‐friendly and circular polarization imaging modes (see Figure 9). The identity line is indicated in blue.

## Data Availability

Data files and code for generating results in Figures 3, 4, 5, 6, 7, 8, 9 and S1, and to set and read data from lab instruments are available at Zenodo (https://doi.org/10.5281/zenodo.14603090).
