# Supplementary material for: Read My Leads: Subject‐Specific RF Hazard Assessment and Mitigation for DBS Implants in MRI
Source: Magn Reson Med. 2025 Nov 24;95(4):2316–30. doi: 10.1002/mrm.70186 (PMC12850618; doi:10.1002/mrm.70186)
Supplement: Supplementary file 1 — Figure S1: Parcellation of the cortex using conventional and implant‐friendly imaging. The brain region volumes are calculated based on Destrieux atlas using implant‐friendly and circular polarization imaging modes (see Figure 9). The identity line is indicated in blue. [file MRM-95-2316-s001.docx]

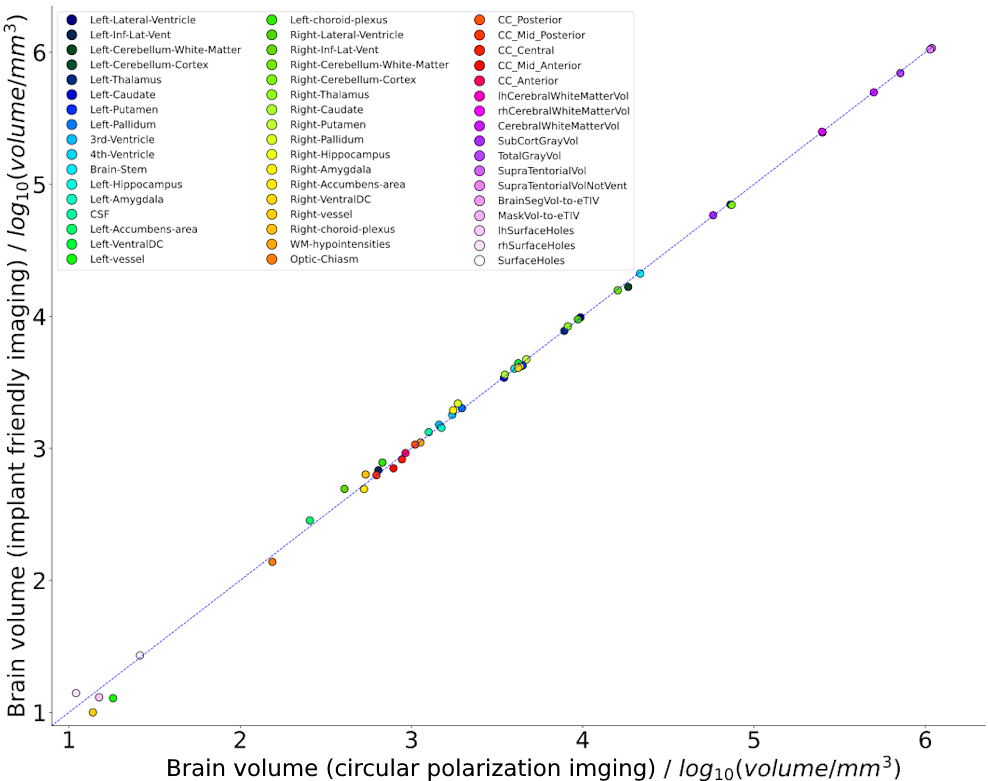


Figure S1: Parcellation of the cortex using conventional and implant-friendly imaging. The brain region volumes are calculated based on Destrieux atlas using implant-friendly and circular polarization imaging modes (see Figure 9). The identity line is indicated in blue.
